# An amino acid metabolism-based seventeen-gene signature correlates with the clinical outcome and immune features in pancreatic cancer

**DOI:** 10.3389/fgene.2023.1084275

**Published:** 2023-06-02

**Authors:** Jie Hao, Cancan Zhou, Zheng Wang, Zhenhua Ma, Zheng Wu, Yi Lv, Rongqian Wu

**Affiliations:** ^1^ Department of Hepatobiliary Surgery, The First Affiliated Hospital of Xi’an Jiaotong University, Xi’an, China; ^**2**^ Institute of Advanced Surgical Technology and Engineering, The First Affiliated Hospital of Xi’an Jiaotong University, Xi’an, China

**Keywords:** pancreatic cancer, amino acid metabolism, genomic alterations, tumor microenvironment, immunotherapy, chemosensitivity, prognosis

## Abstract

**Background:** Pancreatic cancer is an aggressive tumor with a low 5-year survival rate and primary resistance to most therapy. Amino acid (AA) metabolism is highly correlated with tumor growth, crucial to the aggressive biological behavior of pancreatic cancer; nevertheless, the comprehensive predictive significance of genes that regulate AA metabolism in pancreatic cancer remains unknown.

**Methods:** The mRNA expression data downloaded from The Cancer Genome Atlas (TCGA) were derived as the training cohort, and the GSE57495 cohort from Gene Expression Omnibus (GEO) database was applied as the validation cohort. Random survival forest (RSF) and the least absolute shrinkage and selection operator (LASSO) regression analysis were employed to screen genes and construct an AA metabolism-related risk signature (AMRS). Kaplan-Meier analysis and receiver operating characteristic (ROC) curve were performed to assess the prognostic value of AMRS. We performed genomic alteration analysis and explored the difference in tumor microenvironment (TME) landscape associated with KRAS and TP53 mutation in both high- and low-AMRS groups. Subsequently, the relationships between AMRS and immunotherapy and chemotherapy sensitivity were evaluated.

**Results:** A 17-gene AA metabolism-related risk model in the TCGA cohort was constructed according to RSF and LASSO. After stratifying patients into high- and low-AMRS groups based on the optimal cut-off value, we found that high-AMRS patients had worse overall survival (OS) in the training cohort (a median OS: 13.1 months vs. 50.1 months, *p* < 0.0001) and validation cohort (a median OS: 16.2 vs. 30.5 months, p = 1e-04). Genetic mutation analysis revealed that KRAS and TP53 were significantly more mutated in high-AMRS group, and patients with KRAS and TP53 alterations had significantly higher risk scores than those without. Based on the analysis of TME, low-AMRS group displayed significantly higher immune score and more enrichment of T Cell CD8^+^ cells. In addition, high-AMRS-group exhibited higher TMB and significantly lower tumor immune dysfunction and exclusion (TIDE) score and T Cells dysfunction score, which suggested a higher sensitive to immunotherapy. Moreover, high-AMRS group was also more sensitive to paclitaxel, cisplatin, and docetaxel.

**Conclusion:** Overall, we constructed an AA-metabolism prognostic model, which provided a powerful prognostic predictor for the clinical treatment of pancreatic cancer.

## 1 Introduction

Pancreatic cancer is an aggressive malignancy that is the seventh leading cause of cancer mortality worldwide, and is anticipated to be the third-leading cause of cancer deaths by 2025, with approximately 496,000 cases and 466,000 deaths, emphasizing the poor prognosis of patients with pancreatic cancer (Advancing on pancreatic cancer, 2021; Sung et al., 2021). Despite with significant advances in the treatment of pancreatic cancer in the past decade, it remains one of the fatal cancers in 2023 with a 5-year survival rate of only 11% (Stoffel et al., 2023). There are several reasons for the dismal poor prognosis of pancreatic cancer: one reason for this is that 90% of pancreatic cancer cases are diagnosed at advanced stages (Wood et al., 2022) which are typically characterized by nonspecific symptoms. To note, due to the few prevalent genetic mutations (KRAS, CDKN2A, TP53, and SMAD4) in pancreatic cancer, there are few therapeutic drug options. In addition, pancreatic cancer appears to be resistant to conventional treatments such as chemotherapy and radiation (Kleeff et al., 2016). Consequently, it is necessary to investigate a novel biomarker for the early detection and individualized treatment of pancreatic cancer patients.

Metabolism reprogramming has important implications for tumor progression (Pavlova and Thompson, 2016; Wang and Zou, 2020), the citric acid cycle and pentose phosphate pathway are the main central carbon metabolism in cancer metabolism, while recent studies found that non-carbon metabolism, specifically amino acid (AA) metabolism, contributes to cancer proliferation and growth (Lieu et al., 2020). AA, also known as the basic component of proteins, plays a key role as a signaling molecule in regulating energy and metabolic homeostasis and is crucial for a neoplasm to sustain its proliferation (Vettore et al., 2020; Hu and Guo, 2021). Modern research finds that AA metabolism is highly correlated with cancer progression (Wei et al., 2020), the metabolism of AA, especially glutamine, serine, and glycine, have been identified as key metabolic regulators in supporting tumorigenesis and malignancy (Li and Zhang, 2016). Previously study demonstrated that targeting glutamine metabolism could enhance antitumor immunity and decrease tumor growth in breast cancer by promoting the differentiation of myeloid-derived suppressor cells (Oh et al., 2020). Besides, it was reported that glutamine metabolism is highly associated with oncogenic regulation and tumor microenvironment (TME) (Yang et al., 2017). Additionally, certain AA plays important roles in cancer cell proliferation, including arginine, alanine, and tryptophan, primarily by controlling metabolism within the TME (Martínez-Reyes and Chandel, 2021). Furthermore, studies have found that SLC38A9, an arginine-regulated transporter of several essential AAs, was highly correlated with pancreatic cancer, mainly affecting the biological activities of cell macropinocytosis (Wyant et al., 2017). As the relationship between AA metabolism and pancreatic cancer remains unclear, it is urgent to explore effective and reliable biomarkers for pancreatic cancer therapy based on AA metabolism. High-throughput sequencing and machine learning have transformed cancer research by enabling researchers to identify and characterize bioprognostic markers that can predict patient response to treatment and prognosis. The use of high-throughput sequencing technologies such as next-generation sequencing allows for rapid and cost-effective sequencing of large amounts of genetic material. Machine learning algorithms then analyze and interpret the data to identify biomarkers and develop predictive models. The integration of these technologies has led to significant advances in personalized cancer treatment, allowing for more accurate diagnoses, better treatment selection, and improved patient outcomes. Overall, the application of high-throughput sequencing and machine learning has the potential to revolutionize cancer care and improve patient lives (Chi et al., 2022; Zhao et al., 2022; Hao et al., 2023; Zhao et al., 2023).

In this work, to systematically identify the prognosis value of AA-metabolism-related genes in pancreatic cancer, we attempted to construct an AA metabolism-related risk score (AMRS) signature according to random survival forest (RSF) and least absolute shrinkage and selection operator (LASSO). Our study indicated that AMRS could predict the survival ability of pancreatic cancer patients in the TCGA cohort and was validated in an external validation set. In addition, we comprehensively evaluated the genomic alteration, TME landscape, immunotherapy benefits and chemosensitivity between high- and low-AMRS groups. Taken together, our findings demonstrated that AMRS can serve as a novel and reliable prognostic predictor for the treatment of pancreatic cancer patients.

## 2 Materials and methods

### 2.1 Data collection and processing

The TCGA- Pancreatic ductal adenocarcinoma (PDAC) cohort containing expression data and clinical information of 146 PDAC patients (excluding those with M1 disease) was obtained from The Cancer Genome Atlas (TCGA) dataset (https://portal.gdc.cancer.gov) as a training set. The expression data and follow-up information in GSE57495 downloaded from Gene Expression Omnibus (GEO) database (https://www.ncbi.nlm.nih.gov/gds) were used as a validation set. The GSE63557 cohort from GEO and the IMvigor210 cohort from IMvigor210CoreBiologies (http://research-pub.gene.com/IMvigor210CoreBiologies) were selected as two immunotherapy cohorts.

### 2.2 Differential expression of AA metabolism-related genes

Coherently expressed genes of AA metabolism processes were downloaded from the Gene Ontology (GO) (http://geneontology.org/). Then, a total of 428 AA metabolism-related genes were extracted after eliminating the duplicated genes, which were further used as candidate genes for establishing the prognostic model (Supplementary Table S1). The enrichment scores of 428 AA metabolism-related genes for each pancreatic cancer patient were quantified by the Gene Set Variation Analysis (GSVA) package according to single sample Gene Set Enrichment Analysis (ssGSEA), and we obtained the differentially expressed genes (DEGs) that were screened with the threshold set at |log2 fold change| > 0.585 and an adjusted *p*-value <0.05 using the “limma” package.

### 2.3 Construction and external verification of AA metabolism-related risk model

Then, we performed 42 combinations of 6 machine learning algorithms, including the least absolute shrinkage and selection operator (LASSO) regression analysis, Ridge, stepwise Cox, CoxBoost, random survival forest (RSF), elastic network (Enet) based on 10-fold cross-validation to screen out the most valuable AA metabolism-related risk signature (AMRS) with the highest C-index for predicting prognosis. Subsequently, we constructed an AMRS according to RSF and Lasso, with RSF applied to screen out the most valuable AA metabolism-related genes and LASSO employed to obtain the most reliable risk model. Based on the regression coefficient corresponding to the expression level of each gene, the AMRS of each patient was calculated as the formula: risk score = $\sum_{j=1}^{n}{Expr}_{genej}*{Coef}_{genej}$ , the patients in the training cohort were stratified into high- and low-AMRS groups according to the best cut-off. To further verify the AMRS, the same formula was applied for the risk score calculation in the validation cohorts GSE57495. Moreover, we applied the validation of the AMRS in the TCGA pan-cancer cohorts.

### 2.4 Nomogram construction

Based on the AMRS and clinical characteristics (age, gender, T stage, N stage, pathological stage, and histological grade), we performed univariate and multivariate Cox regression analysis. A nomogram was constructed using the “rms” R package according to the clinical characteristics and AMRS score. To further assess the predictive performance of the nomogram, we applied the calibration curves of 12-month, 24-month, and 36-month, and the receiver operating characteristic (ROC) curve was used to evaluate the predictive ability of the Nomogram.

### 2.5 Analysis of AMRS-related biological functions

Based on the TCGA-PDAC cohort, the “limma” R package was employed to obtain DEGs between high- and low-AMRS groups, the criteria were set as FDR <0.05 and |log2FC| ≥ 0.585. We identified the biological functions of DEGs by running a gene set enrichment analysis (GSEA) based on the Molecular Signatures Database (MSigDB, version 7.2) with a total of 50 hallmark pathways obtained from MsigDB. Furthermore, The Kyoto Encyclopedia of Genes and Genomes (KEGG) (Kanehisa et al., 2021) and Gene Ontology (GO) (Harris et al., 2004) analyses were performed using the “cluster Profile” R package.

### 2.6 Mutation landscape underlying the AMRS

The somatic variants with mutation annotation format (MAF) of the TCGA-PDAC dataset were obtained from UCSC Xena (http://xena.ucsc.edu/). Moreover, we used Fisher’s test to compare the difference in the prevalence of mutated genes between high- and low-AMRS groups using the R package “maftools”, and genes with a *p*-value less than 0.05 were classified as differentially mutated genes. In addition, mutually exclusive and co-occurring genes between two groups were identified by pair-wise Fisher’s Exact test with R package “maftools”.

### 2.7 Estimation of tumor microenvironment of AMRS

Based on the gene-expression profiles, the CIBERSORT algorithm that was sensitive to discrimination of 22 human immune cells phenotypes was applied to evaluate the expression level of immune cell infiltration (http://cibersort.stanford.edu/), and the ESTIMATE algorithm was used to compare the immune score between high- and low-AMRS groups. In addition, we compared the differential expression of immune checkpoints between high- and low-AMRS groups.

### 2.8 Evaluation of immunotherapy response of AMRS

Tumor mutational burden (TMB) was used to predict the potential immunotherapy response. Furthermore, we used the GSE63557 dataset and metastatic urothelial tumors dataset IMvigor210 to investigate the correlation between immunotherapy response and AMRS. We processed the data using the R package “IMvigor210CoreBiologies” in the IMvigor210 cohort. In addition, tumor immune dysfunction and exclusion (TIDE) was applied to identify the underlying immune checkpoint blockade response between high- and low-AMRS groups in pancreatic cancer based on the TIDEweb (http://tide.dfci.harvard.edu/).

### 2.9 Prediction of the chemotherapeutic response

Based on the public pharmacological Web portal, Genomics of Drug Sensitivity in Cancer (GDSC) (https://www.cancerrxgene.org/), we estimated the half-maximal inhibitory concentration (IC50) of common chemotherapeutic drugs for pancreatic cancer by “pRRophetic” R package. In addition, we also investigated the chemotherapy response-related pathways by GSEA analysis.

### 2.10 Statistical analysis

The different overall survival (OS) between the low- and high-AMRS groups was evaluated by Kaplan-Meier survival analysis using the Log-rank test, and the R package “timeROC” was performed to draw the ROC curves and to calculate the AUC. The hazard ratios for univariate analyses were calculated using a univariate Cox proportional hazards regression model. Differences between the two groups were evaluated by Wilcoxon and Kruskal-Wallis tests. R software (v.4.1.2) was employed to perform the statistical analyses, and a two-sided *p* < 0.05 was considered statistically significant.

## 3 Results

### 3.1 Expression profile of AA-metabolism-related genes

**FIGURE 1:**
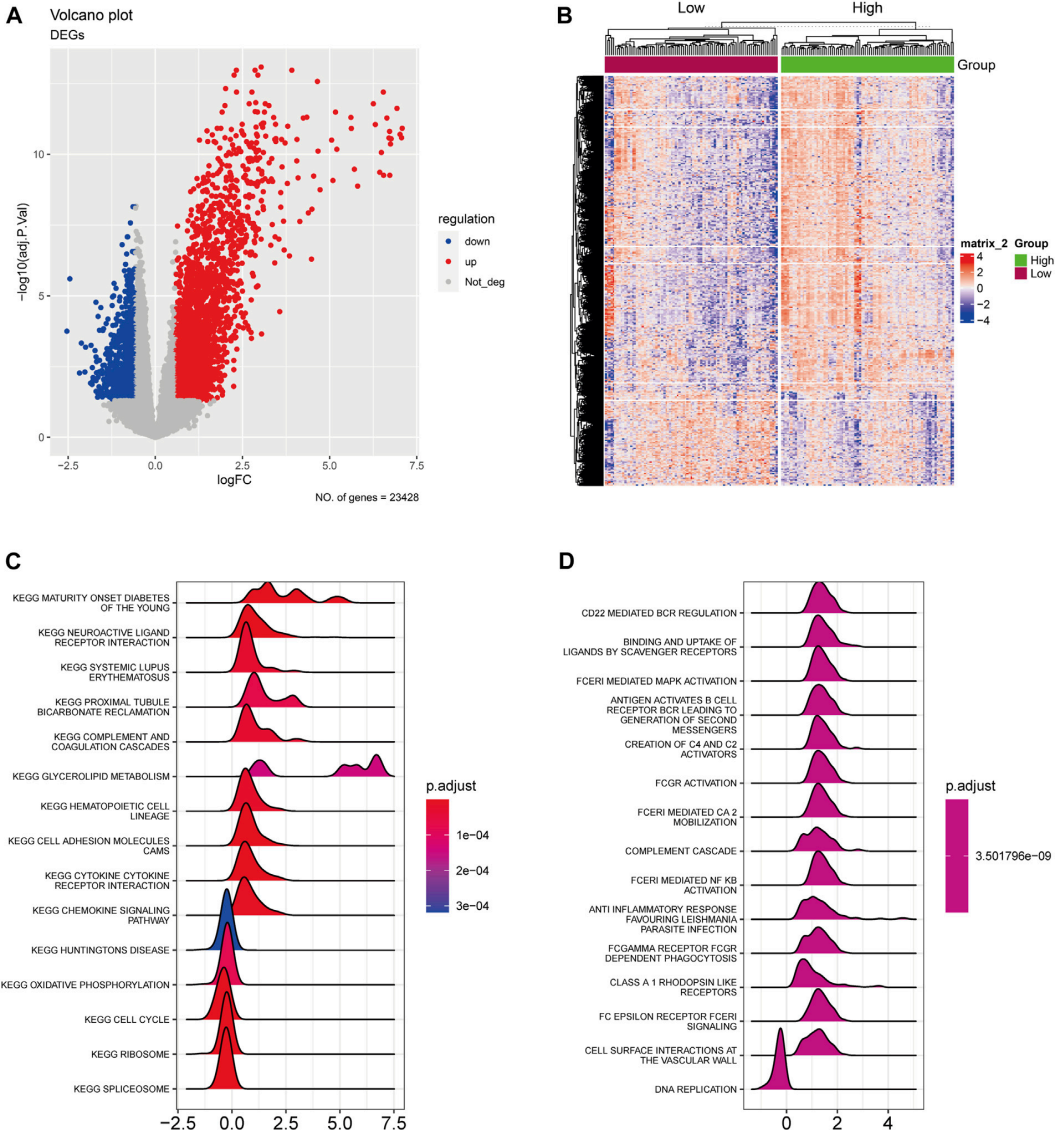
The expression landscape of AA-metabolism-related genes. **(A)** The volcano plot showed downregulated and upregulated AA metabolism-related DEGs. **(B)** The heatmap displayed DEGs were divided into high- and low-expression groups. **(C)** KEGG analysis of DEGs. **(D)** Reactome analysis of DEGs. AA: amino acids, DEGs: differentially expressed genes, KEGG: Kyoto Encyclopedia of Genes and Genomes.

Initially, we identified the AA-metabolism-related DEGs using rigorous selection criteria, which included |logFC|>0.585 and FDR<0.05. Within the TCGA cohort, we discovered 2,771 upregulated DEGs and 823 downregulated DEGs (Figure 1A). Additionally, the pancreatic cancer patients were classified into high- and low-expression groups according to the expression level of those identified DEGs (Figure 1B). The KEGG pathway analysis indicated that those DEGs were significantly correlated with neuroactive ligand receptor interaction, glycerolipid metabolism, cell adhesion molecules cams, and cytokine receptor interaction (Figure 1C). In terms of Reactome analysis, DEGs were mainly associated with CD22 mediated bcr regulation, binding and uptake of ligands by scavenger receptors, and fceri mediated MAPK activation antigen activates B Cell (Figure 1D).

### 3.2 Development and validation of AMRS

**FIGURE 2:**
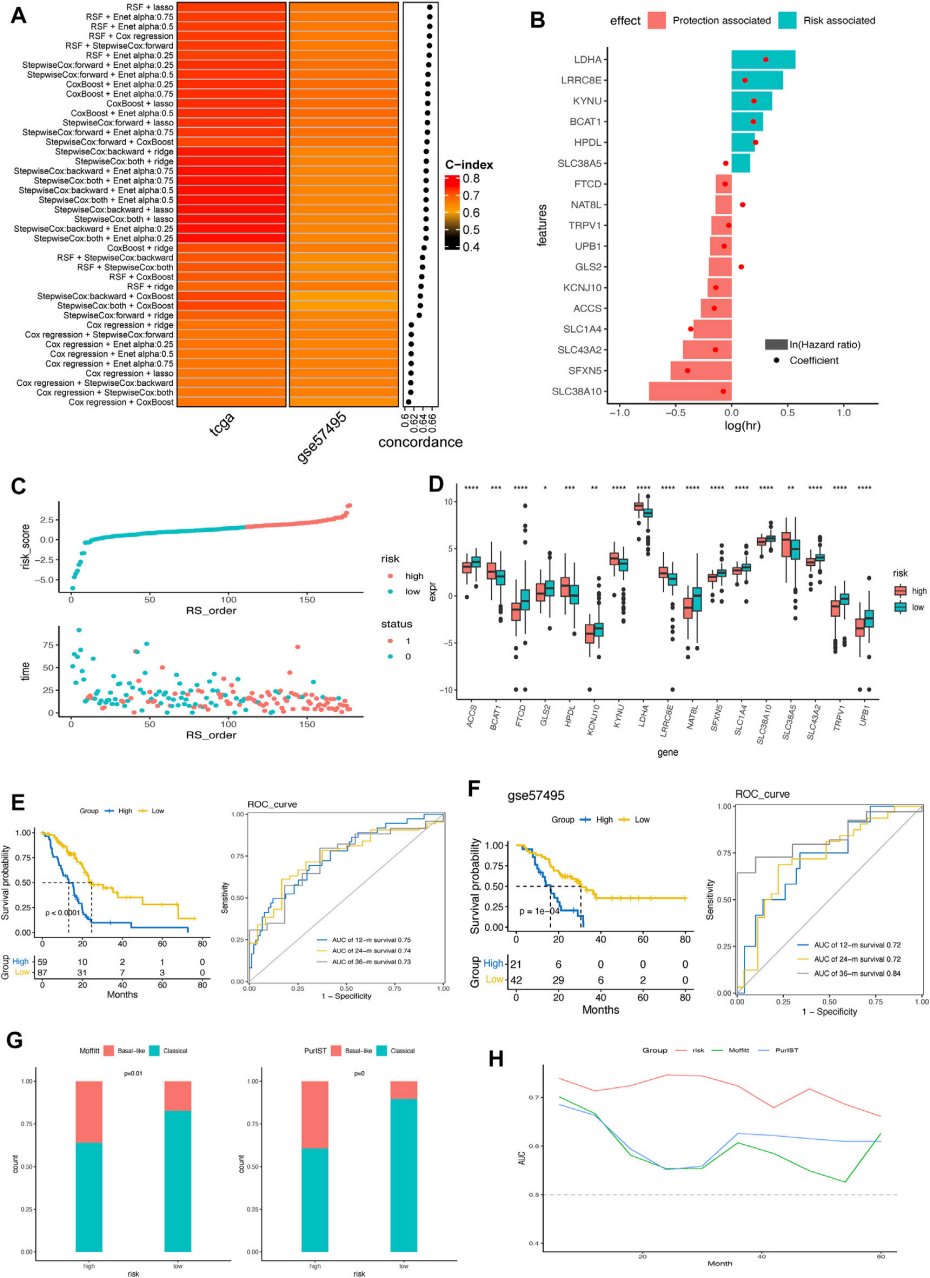
Construction and verification of AMRS in pancreatic cancer. **(A)** 42 combinations of machine learning algorithms of AMRS and the Concordance-index were calculated through TCGA and GSE57495 cohorts. **(B)** Identification of hazard factors and protective factors by multivariate Cox regression analysis. **(C)** Risk score distribution and survival status in TCGA cohort. **(D)** Box plot showing the expression level of 17 genes between high- and low-AMRS group. **(E)** Survival analysis and ROC curve for predicting OS of 12-, 24-, and 36-month in TCGA cohort. **(F)** Kaplan–Meier curve and ROC curve for predicting OS at 12-, 24-, and 36-month in GSE57495 cohort. **(G)** The distribution of Moffitt subtypes and PurIST subtypes in high- and low-AMRS group. **(H)** AUC value for the AMRS, Moffitt subtypes and PurIST subtypes in TCGA cohort. AMRS: amino acid metabolism-related risk score; TCGA: The Cancer Genome Atlas; ROC: receiver operating characteristic; OS: overall survival; AUC: Area Under the ROC Curve.

Based on the 428 AA metabolism-related genes, 6 machine learning algorithms, containing LASSO, Ridge, stepwise Cox, CoxBoost, RSF, and Enet were combined according to 10-fold cross-validation to identify the most robust AMRS with the highest C-index in both the TCGA-PDAC cohort and validating cohort GSE57495 (Figure 2A). Accordingly, an AMRS with the best performance was constructed based on the combined RSF and LASSO algorithms, which collected 17 optimal AA metabolism-related genes, including NAT8L, SLC1A4, SFXN5, LRRC8E, KYNU, TRPV1, KCNJ10, UPB1, FTCD, SLC43A2, HPDL, SLC38A10, LDHA, GLS2, ACCS, SLC38A5, and BCAT1 (Supplementary Table S2). The risk score for each sample was calculated based on the expression level and Cox regression coefficient of these 17 genes as follows:

AMRS score = 0.098× EXP_NAT8L_ - 0.37× EXP_SLC1A4_ - 0.39× EXP_SFXN5_ + 0.12× EXP_KYNU_ -0.026× EXP_TRPV1_ - 0.14× EXP_KCNJ10_–0.067× EXP_UPB1_ - 0.059× EXP_FTCD_ - 0.14× EXP_SLC43A2_ +0.21× EXP_HPDL_ - 0.074× EXP_SLC38A10_ + 0.30× EXP_LDHA_ + 0.085× EXP_GLS2_ - 0.16× EXP_ACCS_ -0.053× EXP_SLC38A5_ + 0.19× EXP_BCAT1_

As shown in Figure 2B, we found that LDHA, LRRC8E, KYNU, BCAT1, HPDL, and SLC38A5 were the risk factors, and the other 11 genes were protective factors according to the multivariate Cox regression analysis. Based on the best cut-off of the AMRS, pancreatic cancer patients in the training cohort were stratified into high- and low-AMRS groups (Figure 2C). Patients with higher tumor stage, lymph node metastasis (N) stage and tumor grades had significantly higher AMRS score (Supplementary Figure S1). We also analyzed the expression profile of the 17 model genes between high- and low-AMRS groups (Figure 2D), the result showed that all the expression level of 17 model genes were significantly differed between high- and low-AMRS groups. The Kaplan-Meier survival curve showed that the OS of the low-AMRS group was significantly longer than the high-AMRS group (a median OS: 13.1 months vs. 50.1 months, *p* < 0.0001, Figure 2E). According to the ROC curve, the AUC values used for the prediction of the 12-month, 24-month, the 36-month OS was 0.75, 0.74, and 0.73, respectively. To further verify the prognostic ability of AMRS, we used external data from GEO database (GSE57495) as a validation cohort. The low-AMRS group showed significantly higher OS than that of the high- AMRS group in GSE57495 (a median OS: 16.2 vs. 30.5 months, p = 1e-04, Figure 2F). The robustness of the AMRS in identifying OS of pancreatic cancer patients was confirmed by AUC values: AUC = 0.75 for 12 months, AUC = 0.72 for 24 months, and AUC = 0.84 for 36-months in GSE57495 (Figure 2F), implying that the AMRS had a good performance to evaluate the prognostic value of pancreatic cancer patients. Moreover, TCGA pan-cancer analysis indicated that AMRS had a prognostic effect on PAAD, KIRP, LGG, CESC, LUAD, SARC, LIHC, HNSC, BLCA, BRCA, and UVM (Supplementary Figure S2). In the meantime, significantly more basal-like samples were enriched in the high- AMRS group by both the Moffitt and PrulST subtypes classification system (Figure 2G). AMRS outperformed these two known subtypes classification system in prediction prognosis of pancreatic cancer patients (Figure 2H).

Based on the protein expression profiles of pancreatic cancer samples collected in the Human Protein Atlas (HPA) database, LDHA, GLS2, and SLC38A10 exhibited higher expression levels in pancreatic cancer (Supplementary Figure S3). Additionally, the mRNA expression levels of LDHA and SLC38A10 were comprehensively high in pancreatic cell lines from the CCLE database (Supplementary Figure S4). Furthermore, we compared the mRNA expression differences of model genes between tumor and normal pancreatic tissues from the GSE62452 dataset. Consistent with the result of the risk and protective genes analysis in Figure 2B, we found that risk genes, such as LDHA and KYNU, had significantly higher expression levels in the tumor tissues (Supplementary Figure S5). In contrast, those protective genes were downregulated in the tumor tissues.

### 3.3 The establishment of a nomogram

**FIGURE 3:**
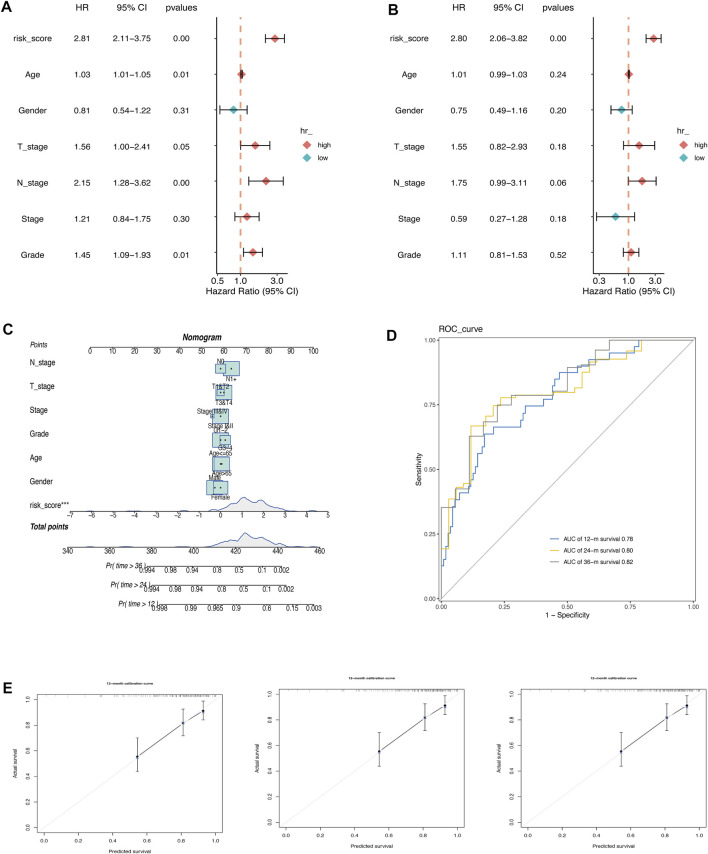
Construction of a Nomogram in pancreatic cancer. **(A)** Univariate Cox regression analysis. **(B)** Multivariate Cox regression analysis. **(C)** Nomogram for predicting 12-, 24-, and 36-month of OS in pancreatic cancer patients in TCGA cohort. **(D)** ROC curve of the nomogram. Calibration curves for predicting the fitness of the nomogram in 12 months, 24 months, and 36 months.E

The prognostic value of AMRS and clinical characteristics, including age, gender, T stage, N stage, pathological stage, and histological grade in predicting OS was investigated using univariate and multivariate Cox regression analyses in the TCGA cohort. The results indicated that AMRS was an independent prognostic factor with the highest hazard ratio (HR) (Figures 3A,B). To improve prognostic accuracy, we developed a nomogram by combining AMRS, T, N, neoplasm stage, grade, age, and gender (Figures 3C,D). The 12-, 24- and 36-months AUC values of the Nomogram were 0.78, 0.80, and 0.82 in the TCGA cohort (Figure 3D). The calibration plots of the nomogram for predicting 12-, 24- and 36-months OS were close to the 45° line, which indicated an accurate prediction of the Nomogram compared to actual observations (Figure 3E).

### 3.4 Association between AMRS and biological functions

**FIGURE 4:**
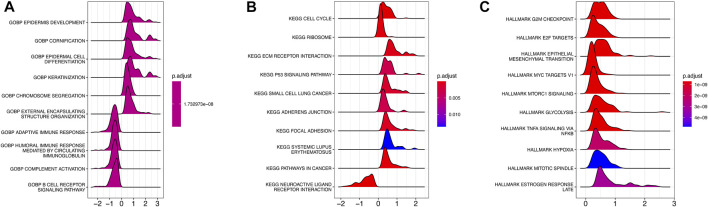
Functional and pathway enrichment analysis of AMRS. **(A)** GO analysis. **(B)** KEGG analysis. **(C)** GSEA analysis. GO: Gene Ontology, GSEA: gene set enrichment analysis.

To investigate the underlying biological function of AMRS in pancreatic cancer patients, we conducted GO and KEGG analyses in the TCGA-PDAC cohort. In the GO analysis, we found that DEGs between high- and low-AMRS groups were mainly enriched in processes related to epidermis development, cornification, epidermal cell differentiation, and keratinization (Figure 4A). Moreover, in terms of KEGG analysis, we observed that cell cycle, ribosome, ECM receptor interaction, and the P53 signaling pathway were the most significantly enriched pathways (Figure 4B). Furthermore, the GSEA analysis using hallmarks revealed that DEGs were enriched in various processes, including G2M checkpoint, E2F targets, epithelial mesenchymal transition, MYC targets V1, mTORC1 signaling, and glycolysis (Figure 4C).

### 3.5 Genomic alterations landscape related to the AMRS

**FIGURE 5:**
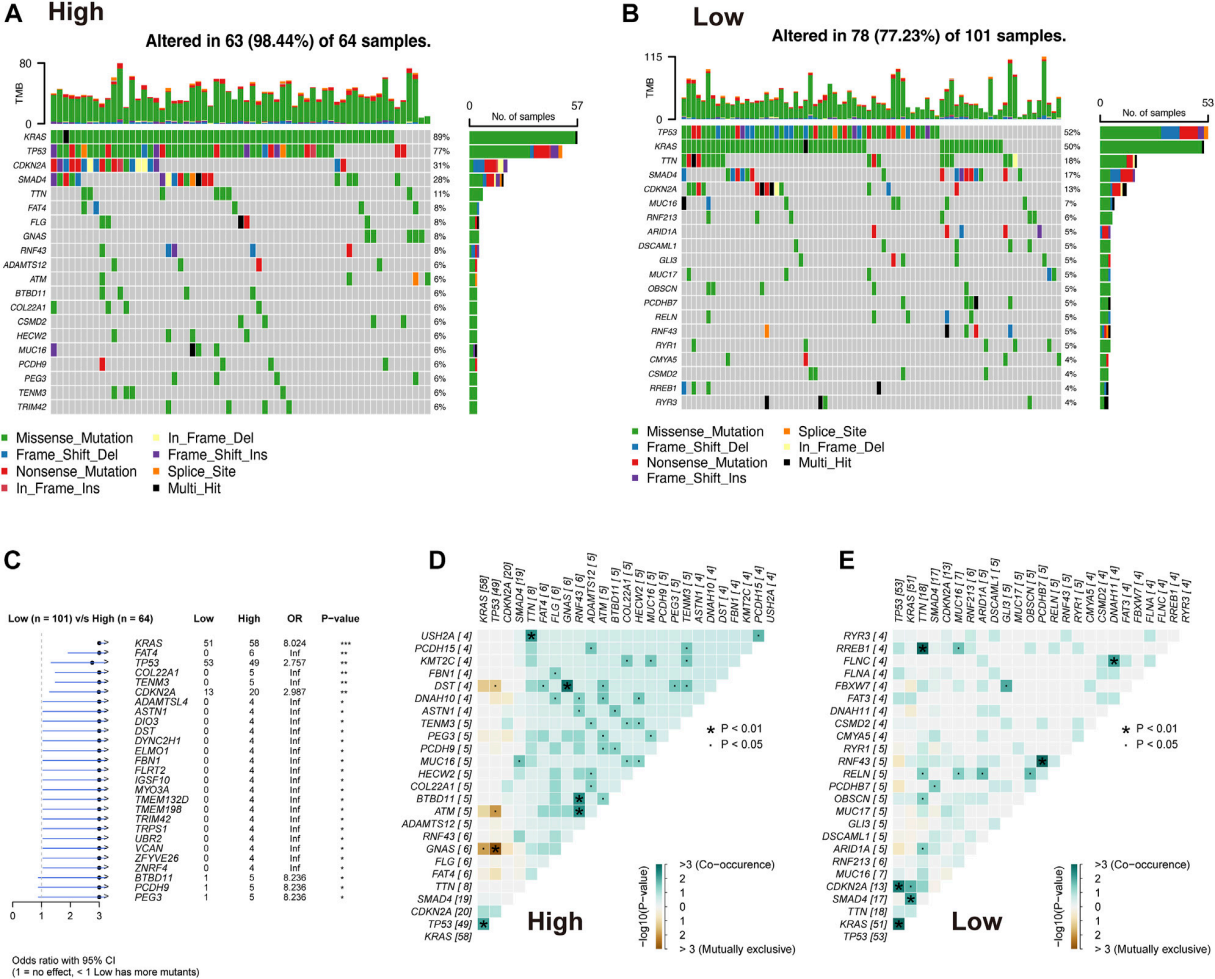
Overview of mutated genes in pancreatic cancer. Waterfall plot of somatic mutation in high AMRS group **(A)** and low AMRS group **(B)**. **(C)** The forest plot shows the 27 genes with the highest differences between the high- and low-AMRS groups. Heat map of mutually exclusive and co-occurring genes in the high-AMRS group **(D)**, and low AMRS groups **(E)**.

**FIGURE 6:**
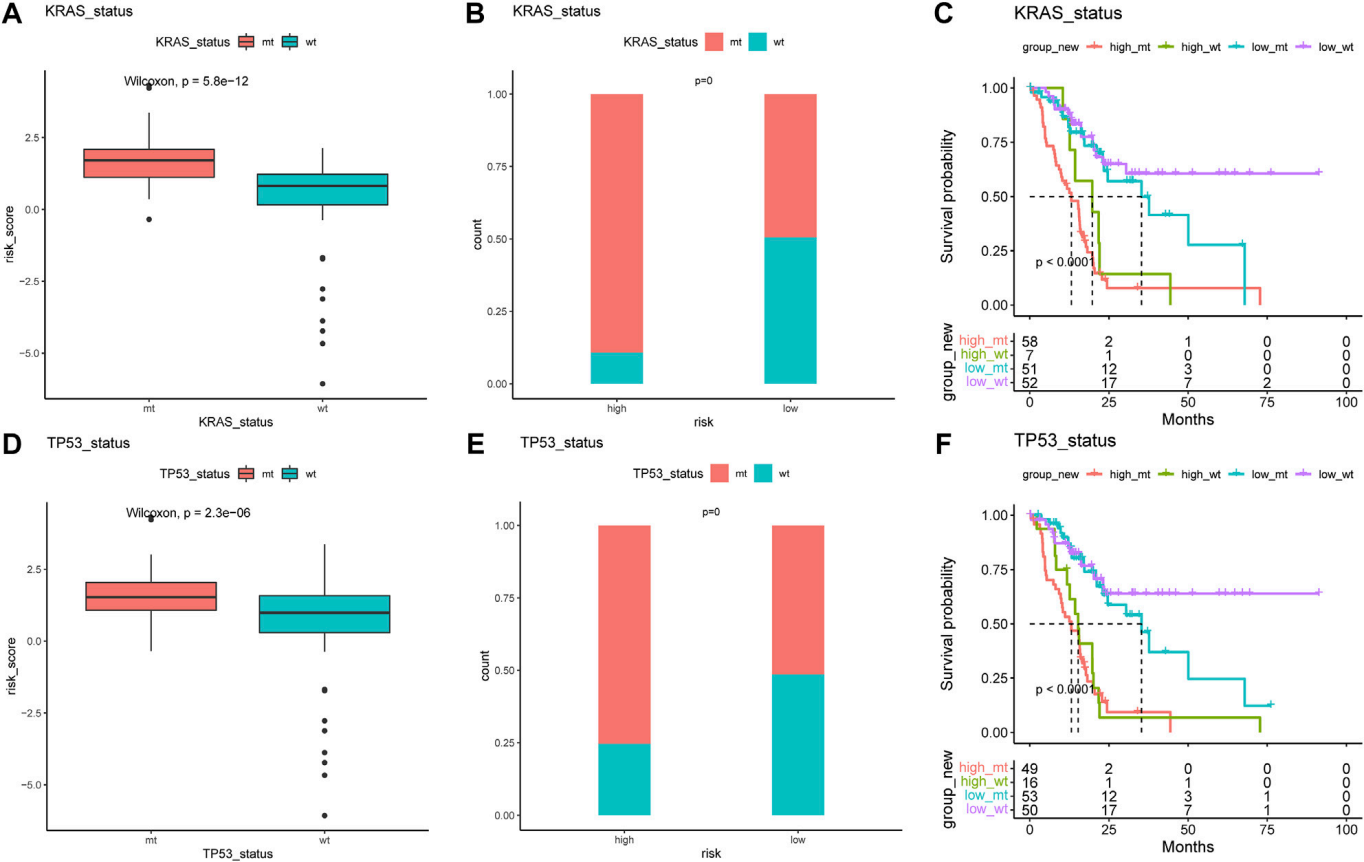
KRAS and TP53 mutation in pancreatic cancer. The risk score of MT and WT in mutation status: KRAS **(A)** and TP53 **(B)**. The proportion of MT and WT between high- and low-AMRS groups in KRAS **(C)** and TP53 **(D)**. **(E)** Survival analysis of MT and WT of KRAS between high- and low-AMRS groups. **(F)** Survival analysis of MT and WT of TP53 between high- and low-AMRS groups. MT: mutation, WT: wild-type.

The difference in the genetic alternations between high- and low-AMRS groups were investigated, and KRAS and TP53 were shown to be the genes with the highest prevalence in both two groups (Figures 5A,B). In addition, the top 20 genes with different mutation frequencies were present in the high- and low-AMRS groups (Figure 5C; Supplementary Table S3). Subsequently, we explored the exclusive and co-occurring genes of the top 25 most frequently mutated genes. In the high-and low-AMRS group, KRAS-TP53 presented the condition of being mutually exclusive, which demonstrated that KRAS-TP53 may have redundant effects in the same pathway (Figures 5D,E). Compared with the group with wild-type (WT) status of KRAS and TP53, the mutation (MT) group had higher risk scores (Figure 6 A&D). Moreover, we noted that in both KRAS and TP53 status, there was a higher proportion of samples with MT in the high-AMRS group and a higher proportion of WT in the low-AMRS group (Figures 6B,E). The mutant status of these two genes statistically significantly stratified patients into high and low-AMRS groups for OS. Specifically, the subgroup of high-AMRS with mt KRAS showed the shortest survival time, while the subgroup of low-AMRS with wt KRAS had significantly prolonged OS (*p* < 0.0001, Figure 6C). On the other hand, we observed that the worst outcomes were seen in patients with high-AMRS and TP53 WT, while the best outcomes were identified in patients of low-AMRS with TP53 WT (*p* < 0.0001, Figure 6F).

### 3.6 Tumor microenvironment analysis of AMRS

**FIGURE 7:**
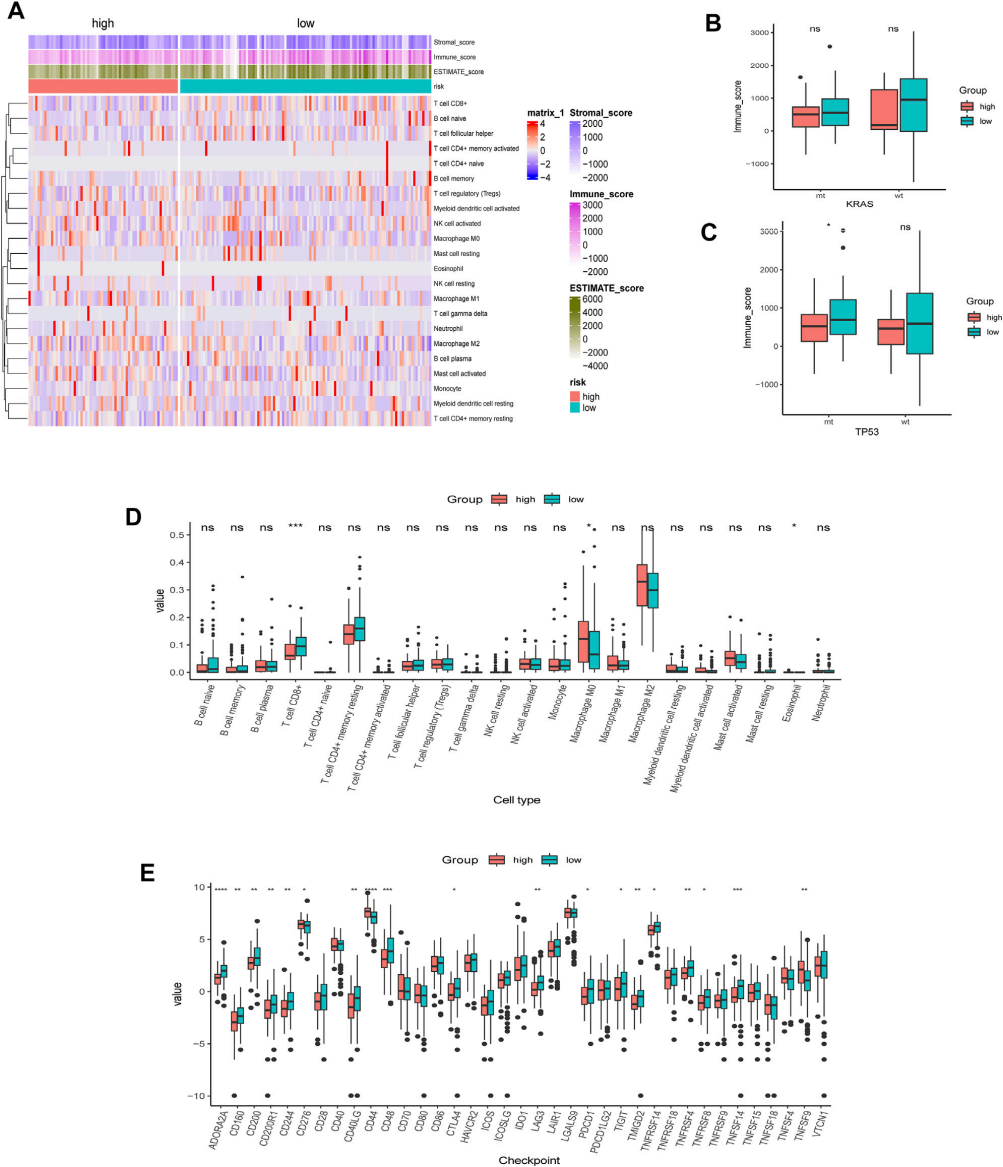
TME analysis of AMRS. **(A)** Heat map of the distribution of immune cell infiltrations in high- and low-AMRS groups. **(B)** Immune score of MT and WT in KRAS status between high- and low-AMRS groups. **(C)** Immune score of MT and WT in P53 status between high- and low-AMRS groups. **(D)** The expression level of 22 tumor infiltrating cells between the two groups. **(E)** Immune checkpoint gene expression level between the two groups. TME: tumor microenvironment.

We first employed the CIBERSORT algorithm and ESTIMATE algorithm to explore the relative abundance of 22 immune infiltrating cells in the high- and low-AMRS groups (Figure 7A). Considering the different frequencies of KRAS and TP53 and their association with the immune system, we investigated the immune scores of patients with or without KRAS/TP53 mutation. We found that a relatively elevated immune score in the low-AMRS of patients regardless of KRAS status (Figure 7B). However, patients with TP53 alteration had significantly higher in the low-AMRS group (Figure 7C). CIBERSORT analysis demonstrated that the higher level of T Cell CD8^+^ in the low-AMRS group, while the immune infiltration level of macrophage M0 was higher in the high-AMRS group (Figure 7D). In addition, the expression levels of immune checkpoint genes, including ADORA2A, CD160, CD200, CD200R1, CD244, CD40LG, CD48, CTLA4, LAG3, PDCD1, TIGIT, TMIGD2, TNFRSF14, TNFRSF4, TNFRSF8, and TNFSF14 were significantly higher in the low-AMRS group; on the contrary, CD276, CD44, and, TNFSF9 were higher in the high-AMRS group (all *p* < 0.05, Figure 7E).

**FIGURE 8:**
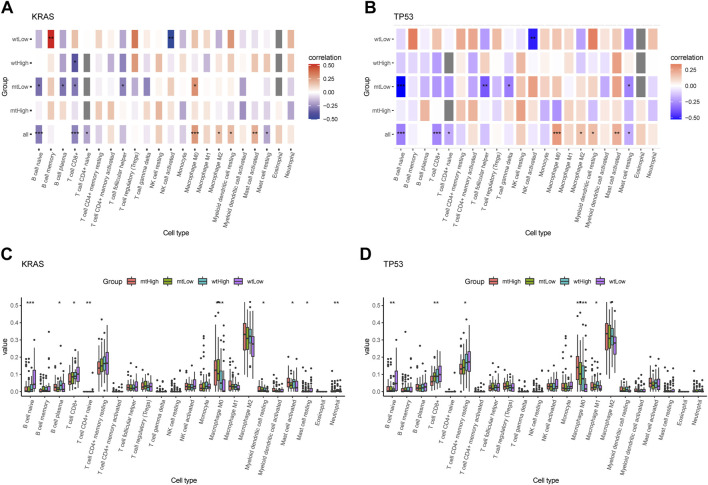
Immune cell infiltration landscape of mutant genes KRAS and TP53. Correlation analysis between 22 tumor infiltrating cells and WT, MT of KRAS **(A)** and TP53 **(B)** in high- and low-AMRS groups. Box plots showing the 22 tumor infiltrating cells expression in MT and WT subgroups of KRAS **(C)** and TP53 **(D)**.

Additionally, the correlation matrix revealed that the infiltrating immune cells were significantly correlated with stratification of both KRAS alteration and AMRS score. Low AMRS score without KRAS alteration had a positive correlation with B Cell memory (*p* < 0.01), and significantly negative correlation with NK cell activated (*p* < 0.01) (Figure 8A). On the other hand, in patients with low AMRS and TP53 alteration,a negative correlation was observed with NK cell activated, B Cell naïve, T Cell follicular helper, T Cell gamma delta, and must cell resting (all *p* < 0.05, Figure 8B). Moreover, high-AMRS with KRAS MT subgroup was characterized by macrophage M0 and mast cell activated, and the low-AMRS with KRAS WT subgroup was distinguished by B Cell naïve, T Cell CD8^+^, and mast cell resting (all *p* < 0.05, Figure 8C). Subsequently, high-AMRS with TP53 MT subgroup had a higher abundance of macrophage M0 and macrophage M2, and the levels of B Cell naïve, T Cell CD8^+^, and T Cell CD4^+^ memory resting were higher in low-AMRS with TP53 WT subgroup (all *p* < 0.05, Figure 8D).

### 3.7 Immunological therapeutic benefits in AMRS

**FIGURE 9:**
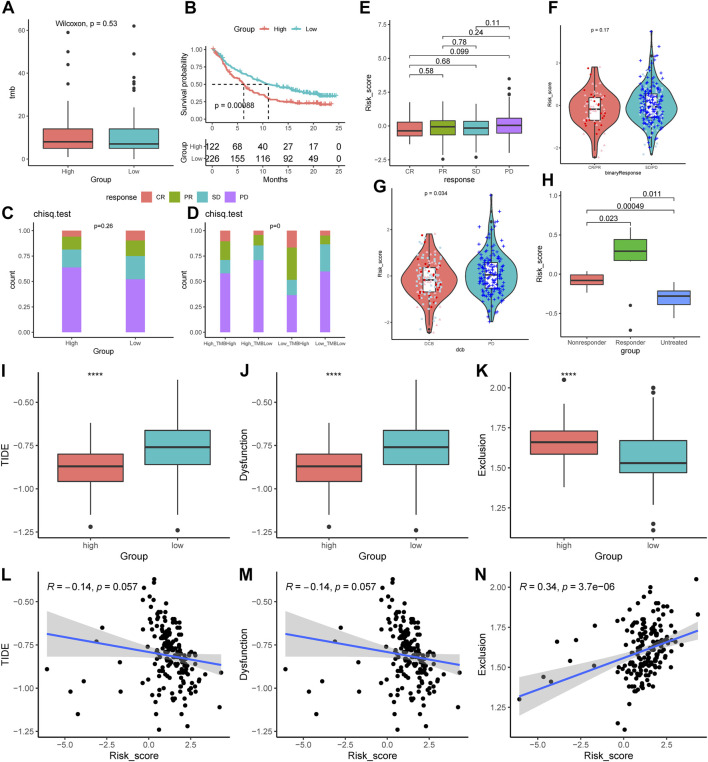
Prediction of the immunological therapeutic benefits in high- and low-AMRS groups. **(A)** Comparison of TMB between high- and low-AMRS groups. **(B)** Kaplan–Meier survival curve in the IMvigor210 cohort. **(C)** The proportion of different immunotherapy responses in high- and low-AMRS groups. **(D)** Analysis of the proportions of four different immunotherapy responses by both TMB groups and the AMRS model. **(E)** Risk score distribution for four different immunotherapy outcomes. **(F)** The difference in AMRS between CR/PR and SD/PD. **(G)** Comparison of risk scores in DCB and PD. **(H)** Difference in risk score among patients with three types of treatment response in the GSE63557 cohort. The distribution of TIDE score **(I)**, T Cells dysfunction score **(J)**, and T Cells exclusion score **(K)**. The relationship between AMRS between TIDE score **(L)**, T Cells dysfunction score **(M)**, and T Cells exclusion score **(N)**. CR/PR: complete response/partial response, SD/PD: stable disease/progressive disease, DCB: durable clinical benefit, TIDE: tumor immune dysfunction and exclusion.

Compared to the low-AMRS groups, the high-AMRS group had a higher TMB, indicating that patients in the high-AMRS group might have a better response to immunotherapy (Figure 9A). Next, we evaluated the predictive ability of AMRS for immunotherapy in patients with metastatic urothelial cancer in the IMvigor210 cohort. Kaplan-Meier curve showed that the low-AMRS group had a better survival trend (*p* = 0.00088, Figure 9B). In addition, we explored the relationship between AMRS and complete response (CR), partial response (PR), stable disease (SD), and progressive disease (PD). Compared with the high-AMRS group, we found that there was no significant difference in patients with different response to immunotherapy (Figures 9C,E,F). The ratio of CR, PR, and SD in the low-AMRS with TMB-high subgroup was significantly higher (Figure 9D). Meanwhile, patients with durable clinical benefit (DCB) had a lower risk score than those with PD (*p* = 0.034, Figure 9G). Moreover, we performed the GSE63557 dataset to investigate the relationship between the AMRS and anti-CTLA-4 therapy, and Figure 9H demonstrated that the responders to anti-CTLA-4 treatment exhibited higher risk score than non-responders and untreated patients (*p* = 0.00049). Besides, we applied TIDE to explore the correlation between AMRS and immune checkpoint blockade response. The results indicated that both the TIDE score and T Cells dysfunction score were lower in the high-AMRS group (both *p* < 0.0001, Figures 9I,J), which demonstrated that the high-AMRS group had a better response to immunotherapy, while the T Cells exclusion score was higher in the high-AMRS group (*p* < 0.0001, Figure 9K). Accordingly, the risk score was negatively related to the TIDE score (Pearson’s correlation, R = −0.14, *p* = 0.057) and T Cells dysfunction score (Pearson’s correlation, R = −0.14, *p* = 0.057), while positively associated with T Cells exclusion score (R = 0.34, *p* = 3.7e-06) (Figures 9L–N).

### 3.8 Prediction for chemotherapy

**FIGURE 10:**
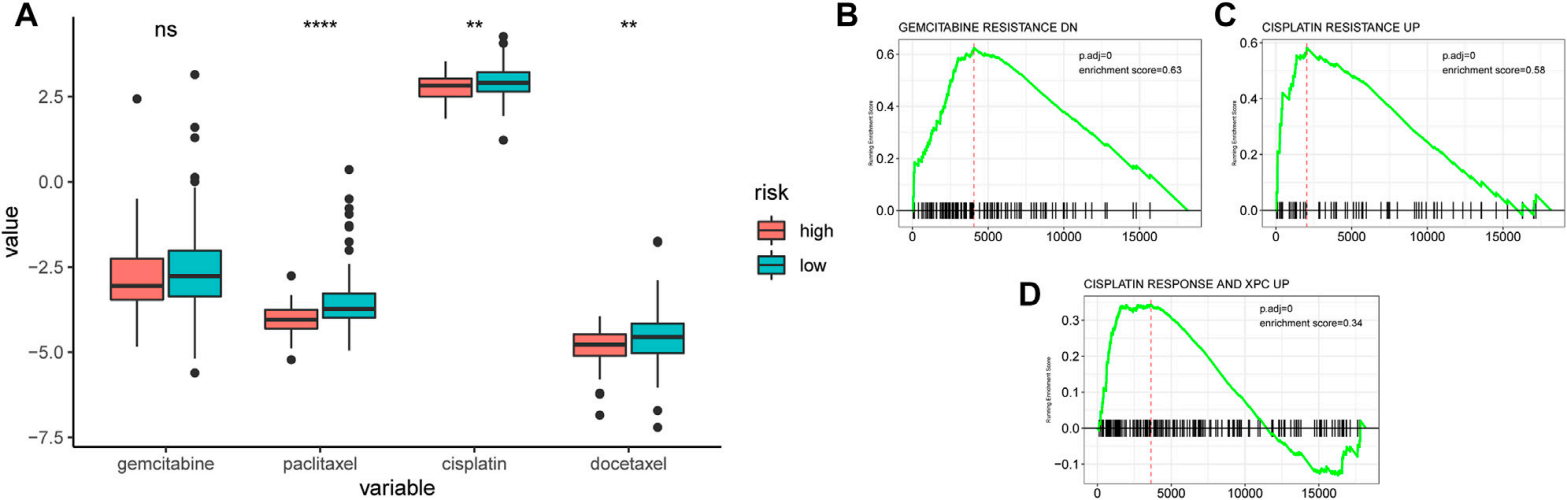
Relationships between AMRS and chemotherapeutic drug sensitivity. **(A)** IC50 comparison of four chemotherapy drugs in high- and low-AMRS groups. Pathways analysis between AMRS and gemcitabine resistance DN **(B)**, cisplatin resistance UP **(C)**, and cisplatin response and XPC UP **(D)**.

The sensitivity of chemotherapeutic drugs in the TCGA-PDAC cohort was evaluated by the GDSC database, the box plot indicated that the IC50 values of paclitaxel, cisplatin, and docetaxel were significantly higher in the low-AMRS group than that in the high-AMRS group (all *p* < 0.01, Figure 10A). Furthermore, pathways associated with a chemotherapeutic response based on the DEGs between the two groups were also assessed by GSEA, which suggested that the AMRS was positively correlated with the pathways of gemcitabine resistance DN, cisplatin resistance UP, and cisplatin response and XPC UP (Figures 10B–D).

## 4 Discussion

In our findings, we comprehensively analyzed the prognostic value of AA-metabolism-related genes in pancreatic cancer and developed an AMRS signature, which provided novel treatment methods for pancreatic cancer. The AMRS was constructed based on 17- AA-metabolism related genes using RSF and LASSO regression analysis in the TCGA. Additionally, we identified the mutation landscape, TME, characteristics of immunotherapy and chemotherapy between high- and low-AMRS groups. This study implied that AMRS can be a reliable biomarker for indicating prognosis potential in pancreatic cancer.

Metabolic reprogramming is a hallmark observed during the development of pancreatic cancer and drug resistance, wherein AA metabolism plays a pivotal role (Liu et al., 2022). AA metabolism is emerging as an important component of cellular metabolic homeostasis, with the potential to accelerate metastasis by tumor cell-extrinsic action and contribute to tumor proliferation and growth (Porporato et al., 2016; Sivanand and Vander Heiden, 2020). Abundant evidence indicates that AA metabolism plays a significant role in the progression, prognosis, and treatment of pancreatic cancer (Mailliard et al., 1995; Qin et al., 2020; Li et al., 2021). Meanwhile, emerging evidence indicates that metabolic plasticity may play a role in reshaping the immune microenvironment and contributing to the therapeutic benefits of immune combination therapy against tumors (Qiu et al., 2023; Xiang et al., 2023). In the current study, a total of 17 genes with prognostic values have been identified, of which 8 genes (SFXN5, LRRC8E, KCNJ10, UPB1, SLC43A2, SLC38A10, ACCS, and SLC38A5) were identified for the first time in pancreatic cancer. Senbabaoglu et al. discovered that NAT8L has an underlying ability to be the biomarker for the diagnosis of neutrally programmed tumors in pancreatic cancer (Senbabaoglu et al., 2020). Moreover, recent research discovered that SLC1A4 could exchange and maintain the concentration of alanine, thereby promoting the proliferation of pancreatic cancer cells (Parker et al., 2020). It was reported that the up-regulation of TRPV1 expression is highly associated with pancreatic cancer, mainly via affecting the biological activity of axonal growth in tumors (Sinha et al., 2014). In addition, HPDL plays a crucial role in the poor prognosis and development of pancreatic cancer, by regulating glutamine metabolism and influencing redox balance (Ye et al., 2020). Besides, high expression of LDHA is correlated with tumor differentiation of pancreatic cancer, which can enhance aerobic glycolysis, resulting in cancer cell proliferation and growth (Shi et al., 2014). In the current study, we constructed a 17-genes prognostic signature based on the AA metabolism-related genes in the TCGA-PDDA, which could provide clinical strategies and individualized treatments for pancreatic cancer patients. In the TCGA cohort, patients in the high-AMRS group had lower OS than those in the low-AMRS group, the prognostic ability of AMRS was also verified in a GEO validation cohort.

Herein, we comprehensively uncovered the biological enrichment pathway of high- and low-AMRS -related genes according to GO, KEGG, and GESA analysis. Numerous studies have found that the ECM receptor interaction pathway is highly related to the prognosis of pancreatic cancer (Xu et al., 2020; Zhuang et al., 2021). Additionally, the G2M checkpoint, as the significant role step in the cell cycle and hallmark of malignant tumors, is associated with the worse OS of pancreatic cancer patients (Oshi et al., 2020). Recent bioinformatics study has demonstrated that E2F targets and MYC targets V1, which are the number of cell proliferation-related pathways, can serve as the predictive biomarker for pancreatic cancer (Oshi et al., 2021). Moreover, p53 signaling can participate in the molecular therapy of pancreatic cancer due to the functions of inducing cell cycle arrest and DNA repair (Ou et al., 2022). Besides, the epithelial mesenchymal transition is involved in the metastasis of cancer by promoting tumor migration, invasiveness, and resistance to apoptosis (Mittal, 2018), which is correlated with the progression of pancreatic cancer (Huang et al., 2017). In addition, the excessive activation of mTORC1 signaling can lead to accelerated development of pancreatic cancer (Revia et al., 2022). Furthermore, glycolysis accelerates pancreatic cancer metastasis, primarily via upregulating the expression of glycolytic enzymes and increasing lactate production (Yang et al., 2020). Collectively, our establishment provided a direction for the investigation of potential biological pathways and the development of clinical treatments for pancreatic cancer.

Pancreatic cancer exhibits high tumor-to-tumor heterogeneity due to genetic mutation and expression diversity (Bear et al., 2020). Oncogenic mutation of KRAS and TP53, which are the main driver genes in pancreatic cancer, is responsible for the activation of multiple cellular processes, such as proliferation, transformation, invasion, and survival (Bournet et al., 2016; Buscail et al., 2020). Moreover, numerous studies have revealed that the KRAS mutation adversely influenced the outcomes of patients with pancreatic cancer (Ogura et al., 2013; Qian et al., 2018). Notability, mutant KRAS enhanced the specific AA intake process named macropinocytosis in pancreatic cancer, resulting in maintaining the AA supply and thus promoting tumor progression (Liu et al., 2019; Qin et al., 2020). In general, KRAS mutation is highly associated with the reprogramming of AA metabolism, which provides nutrition benefits to the tumors (Yang et al., 2017), as well as plays a crucial role in remodeling the immune environment (Kelly and Pearce, 2020). On the other hand, TP53 is associated with a poor prognosis in pancreatic cancer and increased resistance to cancer therapy (Chen et al., 2021), meanwhile, defective TP53 function accelerates cancer cell development and impairs tumor response to DNA-damaging drugs. In particular, TP53 mutation causes AA substitutions in cancer and leads to high expression of TP53 protein mutations in tumors (Wang et al., 2022). In our study, considering the different immune scores and immune cell infiltration between the high- and low-AMRS groups in the MT and WT subgroups of KRAS and TP53, we speculated that AA signature can effectively classify the different TME profiles of mutation status. In addition, CIBERSORT analysis showed that the low-AMRS-group had a higher immune infiltration level of T Cell CD8^+^, which exhibits effective anti-cancer function in the immune system (Sideras et al., 2014). Studies have found that tumor-associated macrophages contribute to the resistance of pancreatic cancer to chemotherapy drugs, mainly via inducing epithelial to mesenchymal transition (Kuwada et al., 2018), which might explain the higher expression level of macrophage M0 in the high-AMRS group. It is reported that checkpoint inhibitors, offer huge benefits for the future treatment of pancreatic cancer (Neoptolemos et al., 2018), which do hold promise for cancer patient s (Nagaraju et al., 2021). Compared with the low-AMRS group, CD276, CD44 and, TNFSF9 exhibited higher in the high-AMRS group. Importantly, CD276 plays a key role in inhibiting T Cell function and displays upregulated expression in solid cancers, which is related to poor prognosis (Picarda et al., 2016). And recent research demonstrated that high expression of CD44 is highly correlated with increased aggressiveness of pancreatic cancer and increased gemcitabine resistance to chemo chemicals (Zhao et al., 2016). Meanwhile, TNFSF9 promotes the metastasis of pancreatic cancer by Wnt/Snail signaling and macrophages M2 (Wu et al., 2021). Overall, we confirmed the AMRS can identify the TME landscape and provide treatment direction for pancreatic cancer patients.

Immunotherapy has exploded in the cancer field over the past decade (The expanding palette of immunotherapy, 2022), which develops into the fourth cancer treatment (Chang et al., 2021), and the immunotherapy based on immune cells and TME also plays an important role in pancreatic cancer (Xu et al., 2018). Our study showed that TMB exhibited higher in the high-AMRS group, due to the TMB is a predictor of survival in the immunotherapy field (Parikh et al., 2019), we believed that the high-AMRS group had a better immunotherapy benefit. In addition, as a member of immune checkpoint molecule, CTLA-4 inhibits the effector T-cell with the highly expressed on Tregs. Studies have found that anti-CTLA-4 therapy brings clinical benefit to cancer patients by depleting Tregs and activating T Cells (Hong and Maleki Vareki, 2022). According to the results from GSE63557 dataset, we discovered that the pancreatic cancer patients with high-risk score response better to anti-CTLA-4 treatment. TIDE, which is performed as a reliable biological indicator to predict the response to immune checkpoint blockade, can identify the factors underlying tumor immune escape mechanisms (Jiang et al., 2018). In our research, TIDE score and T Cells dysfunction score were both lower in the high-AMRS group, and this also proved that the high-AMRS group may benefit more from immunotherapy.

Chemotherapy is one of the most effective clinical treatments for pancreatic cancer (Bednar and Pasca di Magliano, 2020), and common chemotherapy drugs for pancreatic cancer are gemcitabine and cisplatin (Conroy et al., 2018; Hu et al., 2021). Moreover, the chemotherapy drug paclitaxel can improve survival in patients with metastatic pancreatic cancer (Ma and Hidalgo, 2013), as well as docetaxel, has a better therapeutic effect on patients with advanced pancreatic cancer (Lopes and Rocha Lima, 2005). Our research demonstrated that the IC50 values of gemcitabine, paclitaxel, cisplatin and docetaxel were higher in the low-AMRS group, which indicated the chemotherapy was more beneficial in the low-AMRS group. Consequently, we confirmed that AMRS can be applied to predict the response to the chemotherapeutic drug sensitivity of pancreatic cancer patients. The current study has several limitations that should be considered. Firstly, it is a retrospective study that relies on database mining. To confirm the findings, especially the established signature, future prospective studies should be designed and conducted using a large number of pancreatic cancer samples from independent cohorts. Secondly, due to the limited accessibility to databases containing both transcriptome and treatment response data, the usefulness of the established AMRS in tailoring individual therapeutic strategies was solely based on bioinformatic analysis. Further prospective studies should be conducted to validate its practical application. Thirdly, the TCGA database provided a limited number of pancreatic cancer patients with long distant metastasis, making it necessary to investigate the value of the established AMRS in predicting patients with M1 disease in further studies. Finally, while the model genes’ biological function was analyzed using bioinformatic analysis, the precise biological function of these genes in the development of pancreatic cancer remains unknown. Therefore, it is necessary to conduct further *in vitro* and/or *in vivo* experiments to explore this.

In conclusion, our study constructed an AA metabolism-related signature and offered crucial information regarding the genetic mutations, immunological TME landscape, immunotherapy response, and chemosensitivity between high- and low-AMRS groups. Considering that this is the first time AA metabolism-related genes have been studied in pancreatic cancer, we believed that the AMRS model can provide appropriate biological indicators for pancreatic cancer.

## Figures and Tables

**Table:** 

**AA**	Amino acid
**TME**	Tumor microenvironment
**AMRS**	AA metabolism-related risk score
**RSF**	Random survival forest
**LASSO**	Least absolute shrinkage and selection operator
**TCGA**	The Cancer Genome Atlas
**GEO**	Gene Expression Omnibus
**GO**	Gene Ontology
**GSVA**	Gene Set Variation Analysis
**SsGSEA**	Single sample Gene Set Enrichment Analysis
**DEGs**	Differentially expressed genes
**ROC**	Receiver operating characteristic
**GSEA**	Gene set enrichment analysis
**KEGG**	Kyoto Encyclopedia of Genes and Genomes
**TMB**	Tumor mutational burden
**TIDE**	Tumor immune dysfunction and exclusion
**GDSC**	Genomics of Drug Sensitivity in Cancer
**IC50**	Half-maximal inhibitory concentration
**OS**	Overall survival

## Data Availability

The datasets presented in this study can be found in online repositories. The names of the repository/repositories and accession number(s) can be found in the article/Supplementary Material.

## References

[B1] Advancing on pancreatic cancer (2021). Advancing on pancreatic cancer. Nat. Rev. Gastroenterol. Hepatol. 18, 447. 10.1038/s41575-021-00479-5 34188210

[B2] Bear A. S. Vonderheide R. H. O'Hara M. H. (2020). Challenges and opportunities for pancreatic cancer immunotherapy. Cancer Cell 38, 788–802. 10.1016/j.ccell.2020.08.004 32946773 PMC7738380

[B3] Bednar F. Pasca di Magliano M. (2020). Chemotherapy and tumor evolution shape pancreatic cancer recurrence after resection. Cancer Discov. 10, 762–764. 10.1158/2159-8290.CD-20-0359 32482663

[B4] Bournet B. Buscail C. Muscari F. Cordelier P. Buscail L. (2016). Targeting KRAS for diagnosis, prognosis, and treatment of pancreatic cancer: Hopes and realities. Eur. J. Cancer 54, 75–83. 10.1016/j.ejca.2015.11.012 26735353

[B5] Buscail L. Bournet B. Cordelier P. (2020). Role of oncogenic KRAS in the diagnosis, prognosis and treatment of pancreatic cancer. Nat. Rev. Gastroenterol. Hepatol. 17, 153–168. 10.1038/s41575-019-0245-4 32005945

[B6] Chang M. Hou Z. Wang M. Li C. Lin J. (2021). Recent advances in hyperthermia therapy-based synergistic immunotherapy. Adv. Mater 33, e2004788. 10.1002/adma.202004788 33289219

[B7] Chen X. Lin X. Shen Q. Qian X. (2021). Combined spiral transformation and model-driven multi-modal deep learning scheme for automatic prediction of TP53 mutation in pancreatic cancer. IEEE Trans. Med. Imaging 40, 735–747. 10.1109/TMI.2020.3035789 33147142

[B8] Chi H. Peng G. Wang R. Yang F. Xie X. Zhang J. (2022). Cuprotosis programmed-cell-death-related lncRNA signature predicts prognosis and immune landscape in PAAD patients. Cells 11, 3436. 10.3390/cells11213436 36359832 PMC9658590

[B9] Conroy T. Hammel P. Hebbar M. Ben Abdelghani M. Wei A. C. Raoul J. L. (2018). FOLFIRINOX or gemcitabine as adjuvant therapy for pancreatic cancer. N. Engl. J. Med. 379, 2395–2406. 10.1056/nejmoa1809775 30575490

[B10] The expanding palette of immunotherapy research. Nat. Cancer 3 (2022) 651. 35764742 10.1038/s43018-022-00410-0

[B11] Hao C. Jinyan Y. Gaoge P. Jinhao Z. Guobin S. Xixi X. (2023). Circadian rhythm-related genes index: A predictor for HNSCC prognosis, immunotherapy efficacy, and chemosensitivity. Front. Immunol. 14, 1091218. 10.3389/fimmu.2023.1091218 36969232 PMC10036372

[B12] Harris M. A. Clark J. Ireland A. Lomax J. Ashburner M. Foulger R. (2004). The Gene Ontology (GO) database and informatics resource. Nucleic Acids Res. 32, D258–D261. 10.1093/nar/gkh036 14681407 PMC308770

[B13] Hong M. M. Y. Maleki Vareki S. (2022). Addressing the elephant in the immunotherapy room: Effector T-cell priming versus depletion of regulatory T-cells by anti-CTLA-4 therapy. Cancers (Basel) 14, 1580. 10.3390/cancers14061580 35326731 PMC8946681

[B14] Hu F. F. Liu C. J. Liu L. L. Zhang Q. Guo A. Y. (2021). Expression profile of immune checkpoint genes and their roles in predicting immunotherapy response. Brief. Bioinform 22, bbaa176. 10.1093/bib/bbaa176 32814346

[B15] Hu X. Guo F. (2021). Amino acid sensing in metabolic homeostasis and health. Endocr. Rev. 42, 56–76. 10.1210/endrev/bnaa026 33053153

[B16] Huang J. Mei H. Tang Z. Li J. Zhang X. Lu Y. (2017). Triple-amiRNA VEGFRs inhibition in pancreatic cancer improves the efficacy of chemotherapy through EMT regulation. J. Control Release 245, 1–14. 10.1016/j.jconrel.2016.11.024 27889393

[B17] Jiang P. Gu S. Pan D. Fu J. Sahu A. Hu X. (2018). Signatures of T cell dysfunction and exclusion predict cancer immunotherapy response. Nat. Med. 24, 1550–1558. 10.1038/s41591-018-0136-1 30127393 PMC6487502

[B18] Kanehisa M. Furumichi M. Sato Y. Ishiguro-Watanabe M. Tanabe M. (2021). Kegg: Integrating viruses and cellular organisms. Nucleic Acids Res. 49, D545–d551. 10.1093/nar/gkaa970 33125081 PMC7779016

[B19] Kelly B. Pearce E. L. (2020). Amino assets: How amino acids Support immunity. Cell Metab. 32, 154–175. 10.1016/j.cmet.2020.06.010 32649859

[B20] Kleeff J. Korc M. Apte M. La Vecchia C. Johnson C. D. Biankin A. V. (2016). Pancreatic cancer. Nat. Rev. Dis. Prim. 2, 16022. 10.1038/nrdp.2016.22 27158978

[B21] Kuwada K. Kagawa S. Yoshida R. Sakamoto S. Ito A. Watanabe M. (2018). The epithelial-to-mesenchymal transition induced by tumor-associated macrophages confers chemoresistance in peritoneally disseminated pancreatic cancer. J. Exp. Clin. Cancer Res. 37, 307. 10.1186/s13046-018-0981-2 30537992 PMC6288926

[B22] Li J. Y. Sun F. Yang C. L. Zhou H. F. Gao M. Zhang Q. (2021). GEO data mining and TCGA analysis reveal altered branched chain amino acid metabolism in pancreatic cancer patients. Aging (Albany NY) 13, 11907–11918. 10.18632/aging.202892 33882453 PMC8109144

[B23] Li Z. Zhang H. (2016). Reprogramming of glucose, fatty acid and amino acid metabolism for cancer progression. Cell Mol. Life Sci. 73, 377–392. 10.1007/s00018-015-2070-4 26499846 PMC11108301

[B24] Lieu E. L. Nguyen T. Rhyne S. Kim J. (2020). Amino acids in cancer. Exp. Mol. Med. 52, 15–30. 10.1038/s12276-020-0375-3 31980738 PMC7000687

[B25] Liu C. Li C. Liu Y. (2022). The role of metabolic reprogramming in pancreatic cancer chemoresistance. Front. Pharmacol. 13, 1108776. 10.3389/fphar.2022.1108776 36699061 PMC9868425

[B26] Liu H. Sun M. Liu Z. Kong C. Kong W. Ye J. (2019). KRAS-enhanced macropinocytosis and reduced FcRn-mediated recycling sensitize pancreatic cancer to albumin-conjugated drugs. J. Control Release 296, 40–53. 10.1016/j.jconrel.2019.01.014 30653981

[B27] Lopes G. Rocha Lima C. M. (2005). Docetaxel in the management of advanced pancreatic cancer. Semin. Oncol. 32, S10–S23. 10.1053/j.seminoncol.2005.04.003 16015551

[B28] Ma W. W. Hidalgo M. (2013). The winning formulation: The development of paclitaxel in pancreatic cancer. Clin. Cancer Res. 19, 5572–5579. 10.1158/1078-0432.CCR-13-1356 23918602

[B29] Mailliard M. E. Stevens B. R. Mann G. E. (1995). Amino acid transport by small intestinal, hepatic, and pancreatic epithelia. Gastroenterology 108, 888–910. 10.1016/0016-5085(95)90466-2 7875494

[B30] Martínez-Reyes I. Chandel N. S. (2021). Cancer metabolism: Looking forward. Nat. Rev. Cancer 21, 669–680. 10.1038/s41568-021-00378-6 34272515

[B31] Mittal V. (2018). Epithelial mesenchymal transition in tumor metastasis. Annu. Rev. Pathol. 13, 395–412. 10.1146/annurev-pathol-020117-043854 29414248

[B32] Nagaraju G. P. Malla R. R. Basha R. Motofei I. G. (2021). Contemporary clinical trials in pancreatic cancer immunotherapy targeting PD-1 and PD-L1. Semin. Cancer Biol. 86, 616–621. 10.1016/j.semcancer.2021.11.003 34774995

[B33] Neoptolemos J. P. Kleeff J. Michl P. Costello E. Greenhalf W. Palmer D. H. (2018). Therapeutic developments in pancreatic cancer: Current and future perspectives. Nat. Rev. Gastroenterol. Hepatol. 15, 333–348. 10.1038/s41575-018-0005-x 29717230

[B34] Ogura T. Yamao K. Hara K. Mizuno N. Hijioka S. Imaoka H. (2013). Prognostic value of K-ras mutation status and subtypes in endoscopic ultrasound-guided fine-needle aspiration specimens from patients with unresectable pancreatic cancer. J. Gastroenterol. 48, 640–646. 10.1007/s00535-012-0664-2 22983505

[B35] Oh M. H. Sun I. H. Zhao L. Leone R. D. Sun I. M. Xu W. (2020). Targeting glutamine metabolism enhances tumor-specific immunity by modulating suppressive myeloid cells. J. Clin. Investig. 130, 3865–3884. 10.1172/JCI131859 32324593 PMC7324212

[B36] Oshi M. Newman S. Tokumaru Y. Yan L. Matsuyama R. Endo I. (2020). High G2M pathway score pancreatic cancer is associated with worse survival, particularly after margin-positive (R1 or R2) resection. Cancers (Basel) 12, 2871. 10.3390/cancers12102871 33036243 PMC7599494

[B37] Oshi M. Patel A. Le L. Tokumaru Y. Yan L. Matsuyama R. (2021). G2M checkpoint pathway alone is associated with drug response and survival among cell proliferation-related pathways in pancreatic cancer. Am. J. Cancer Res. 11, 3070–3084. 34249445 PMC8263638

[B38] Ou A. Zhao X. Lu Z. (2022). The potential roles of p53 signaling reactivation in pancreatic cancer therapy. Biochim. Biophys. Acta Rev. Cancer 1877, 188662. 10.1016/j.bbcan.2021.188662 34861354

[B39] Parikh K. Huether R. White K. Hoskinson D. Dong H. Adjei A. A. (2019). Overestimation of tumor mutational burden (TMB) using algorithms compared to germline subtraction. J. Clin. Oncol. 37, 2621. 10.1200/jco.2019.37.15_suppl.2621

[B40] Parker S. J. Amendola C. R. Hollinshead K. E. R. Yu Q. Yamamoto K. Encarnación-Rosado J. (2020). Selective alanine transporter utilization creates a targetable metabolic niche in pancreatic cancer. Cancer Discov. 10, 1018–1037. 10.1158/2159-8290.CD-19-0959 32341021 PMC7334074

[B41] Pavlova N. N. Thompson C. B. (2016). The emerging hallmarks of cancer metabolism. Cell Metab. 23, 27–47. 10.1016/j.cmet.2015.12.006 26771115 PMC4715268

[B42] Picarda E. Ohaegbulam K. C. Zang X. (2016). Molecular pathways: Targeting B7-H3 (CD276) for human cancer immunotherapy. Clin. Cancer Res. 22, 3425–3431. 10.1158/1078-0432.CCR-15-2428 27208063 PMC4947428

[B43] Porporato P. E. Payen V. L. Baselet B. Sonveaux P. (2016). Metabolic changes associated with tumor metastasis, part 2: Mitochondria, lipid and amino acid metabolism. Cell Mol. Life Sci. 73, 1349–1363. 10.1007/s00018-015-2100-2 26646069 PMC11108268

[B44] Qian Z. R. Rubinson D. A. Nowak J. A. Morales-Oyarvide V. Dunne R. F. Kozak M. M. (2018). Association of alterations in main driver genes with outcomes of patients with resected pancreatic ductal adenocarcinoma. JAMA Oncol. 4, e173420. 10.1001/jamaoncol.2017.3420 29098284 PMC5844844

[B45] Qin C. Yang G. Yang J. Ren B. Wang H. Chen G. (2020). Metabolism of pancreatic cancer: Paving the way to better anticancer strategies. Mol. Cancer 19, 50. 10.1186/s12943-020-01169-7 32122374 PMC7053123

[B46] Qiu J. Feng M. Yang G. Cao Z. Liu Y. You L. (2023). mTOR inhibitor, gemcitabine and PD-L1 antibody blockade combination therapy suppresses pancreatic cancer progression via metabolic reprogramming and immune microenvironment remodeling in Trp53^flox/+^LSL-Kras^G12D/+^Pdx-1-Cre murine models. Cancer Lett. 554, 216020. 10.1016/j.canlet.2022.216020 36442772

[B47] Revia S. Seretny A. Wendler L. Banito A. Eckert C. Breuer K. (2022). Histone H3K27 demethylase KDM6A is an epigenetic gatekeeper of mTORC1 signalling in cancer. Gut 71, 1613–1628. 10.1136/gutjnl-2021-325405 34509979 PMC9279849

[B48] Senbabaoglu Y. Calviello A. Munoz G. Bourgon R. Turley S. Moussion C. (2020). 667 Integrated molecular characterization and therapeutic vulnerabilities of neurally programmed tumors across 33 human indications. J. Immunother. Cancer 8, A400.

[B49] Shi M. Cui J. Du J. Wei D. Jia Z. Zhang J. (2014). A novel KLF4/LDHA signaling pathway regulates aerobic glycolysis in and progression of pancreatic cancer. Clin. Cancer Res. 20, 4370–4380. 10.1158/1078-0432.CCR-14-0186 24947925 PMC4134726

[B50] Sideras K. Braat H. Kwekkeboom J. van Eijck C. H. Peppelenbosch M. P. Sleijfer S. (2014). Role of the immune system in pancreatic cancer progression and immune modulating treatment strategies. Cancer Treat. Rev. 40, 513–522. 10.1016/j.ctrv.2013.11.005 24315741

[B51] Sinha S. Fu Y. Y. Yang I. H. Kulkarni S. Lee A. M. Joo M. G. (2014). Tu1903 pancreatic cancer and sensory nerves: Characterization of the role of TRPV1 leading to axonal and cancer growth using a novel microfluidic dual chamber system. Gastroenterology 146, 868. 10.1016/s0016-5085(14)63158-9 24468181

[B52] Sivanand S. Vander Heiden M. G. (2020). Emerging roles for branched-chain amino acid metabolism in cancer. Cancer Cell 37, 147–156. 10.1016/j.ccell.2019.12.011 32049045 PMC7082774

[B53] Stoffel E. Brand R. Goggins M. (2023). Gastroenterology.Pancreatic cancer: Changing epidemiology and new approaches to risk assessment, early detection, and prevention 10.1053/j.gastro.2023.02.012 PMC10243302 36804602

[B54] Sung H. Ferlay J. Siegel R. L. Laversanne M. Soerjomataram I. Jemal A. (2021). Global cancer statistics 2020: GLOBOCAN estimates of incidence and mortality worldwide for 36 cancers in 185 countries. CA Cancer J. Clin. 71, 209–249. 10.3322/caac.21660 33538338

[B55] Vettore L. Westbrook R. L. Tennant D. A. (2020). New aspects of amino acid metabolism in cancer. Br. J. Cancer 122, 150–156. 10.1038/s41416-019-0620-5 31819187 PMC7052246

[B56] Wang W. Zou W. (2020). Amino acids and their transporters in T cell immunity and cancer therapy. Mol. Cell 80, 384–395. 10.1016/j.molcel.2020.09.006 32997964 PMC7655528

[B57] Wang Z. Strasser A. Kelly G. L. (2022). Should mutant TP53 be targeted for cancer therapy? Cell Death Differ. 29, 911–920. 10.1038/s41418-022-00962-9 35332311 PMC9091235

[B58] Wei Z. Liu X. Cheng C. Yu W. Yi P. (2020). Metabolism of amino acids in cancer. Front. Cell Dev. Biol. 8, 603837. 10.3389/fcell.2020.603837 33511116 PMC7835483

[B59] Wood L. D. Canto M. I. Jaffee E. M. Simeone D. M. (2022). Pancreatic cancer: Pathogenesis, screening, diagnosis, and treatment. Gastroenterology 163, 386–402.e1. 10.1053/j.gastro.2022.03.056 35398344 PMC9516440

[B60] Wu J. Wang Y. Yang Y. Liu F. Chen J. Jiang Z. (2021). TNFSF9 promotes metastasis of pancreatic cancer through Wnt/Snail signaling and M2 polarization of macrophages. Aging (Albany NY) 13, 21571–21586. 10.18632/aging.203497 34517345 PMC8457569

[B61] Wyant G. A. Abu-Remaileh M. Wolfson R. L. Chen W. W. Freinkman E. Danai L. V. (2017). mTORC1 activator SLC38A9 is required to efflux essential amino acids from lysosomes and use protein as a nutrient. Cell 171, 642–654. 10.1016/j.cell.2017.09.046 29053970 PMC5704964

[B62] Xiang H. Yang R. Tu J. Xi Y. Yang S. Lv L. (2023). Metabolic reprogramming of immune cells in pancreatic cancer progression. Biomed. Pharmacother. = Biomedecine Pharmacother. 157, 113992. 10.1016/j.biopha.2022.113992 36395610

[B63] Xu J. S. Liao K. L. Wang X. He J. Wang X. Z. (2020). Combining bioinformatics techniques to explore the molecular mechanisms involved in pancreatic cancer metastasis and prognosis. J. Cell Mol. Med. 24, 14128–14138. 10.1111/jcmm.16023 33164330 PMC7754005

[B64] Xu J. W. Wang L. Cheng Y. G. Zhang G. Y. Hu S. Y. Zhou B. (2018). Immunotherapy for pancreatic cancer: A long and hopeful journey. Cancer Lett. 425, 143–151. 10.1016/j.canlet.2018.03.040 29605510

[B65] Yang J. Ren B. Yang G. Wang H. Chen G. You L. (2020). The enhancement of glycolysis regulates pancreatic cancer metastasis. Cell Mol. Life Sci. 77, 305–321. 10.1007/s00018-019-03278-z 31432232 PMC11104916

[B66] Yang L. Venneti S. Nagrath D. (2017). Glutaminolysis: A hallmark of cancer metabolism. Annu. Rev. Biomed. Eng. 19, 163–194. 10.1146/annurev-bioeng-071516-044546 28301735

[B67] Ye X. Wei X. Liao J. Chen P. Li X. Chen Y. (2020). 4-Hydroxyphenylpyruvate dioxygenase-like protein promotes pancreatic cancer cell progression and is associated with glutamine-mediated redox balance. Front. Oncol. 10, 617190. 10.3389/fonc.2020.617190 33537239 PMC7848781

[B68] Zhao S. Chen C. Chang K. Karnad A. Jagirdar J. Kumar A. P. (2016). CD44 expression level and isoform contributes to pancreatic cancer cell plasticity, invasiveness, and response to therapy. Clin. Cancer Res. 22, 5592–5604. 10.1158/1078-0432.CCR-15-3115 27267855 PMC5143222

[B69] Zhao S. Chi H. Ji W. He Q. Lai G. Peng G. (2022). A bioinformatics-based analysis of an anoikis-related gene signature predicts the prognosis of patients with low-grade gliomas. Brain Sci. 12, 1349. 10.3390/brainsci12101349 36291283 PMC9599312

[B70] Zhao S. Chi H. Yang Q. Chen S. Wu C. Lai G. (2023). Identification and validation of neurotrophic factor-related gene signatures in glioblastoma and Parkinson's disease. Front. Immunol. 14, 1090040. 10.3389/fimmu.2023.1090040 36825022 PMC9941742

[B71] Zhuang H. Chen X. Dong F. Zhang Z. Zhou Z. Ma Z. (2021). Prognostic values and immune suppression of the S100A family in pancreatic cancer. J. Cell Mol. Med. 25, 3006–3018. 10.1111/jcmm.16343 33580614 PMC7957204

