# Supplementary material for: An amino acid metabolism-based seventeen-gene signature correlates with the clinical outcome and immune features in pancreatic cancer
Source: Front Genet. 2023 Jun 2;14:1084275. doi: 10.3389/fgene.2023.1084275 (PMC10272610; doi:10.3389/fgene.2023.1084275)
Supplement: Supplementary file 4 [file Table4.DOCX]

Supplementary Material

**
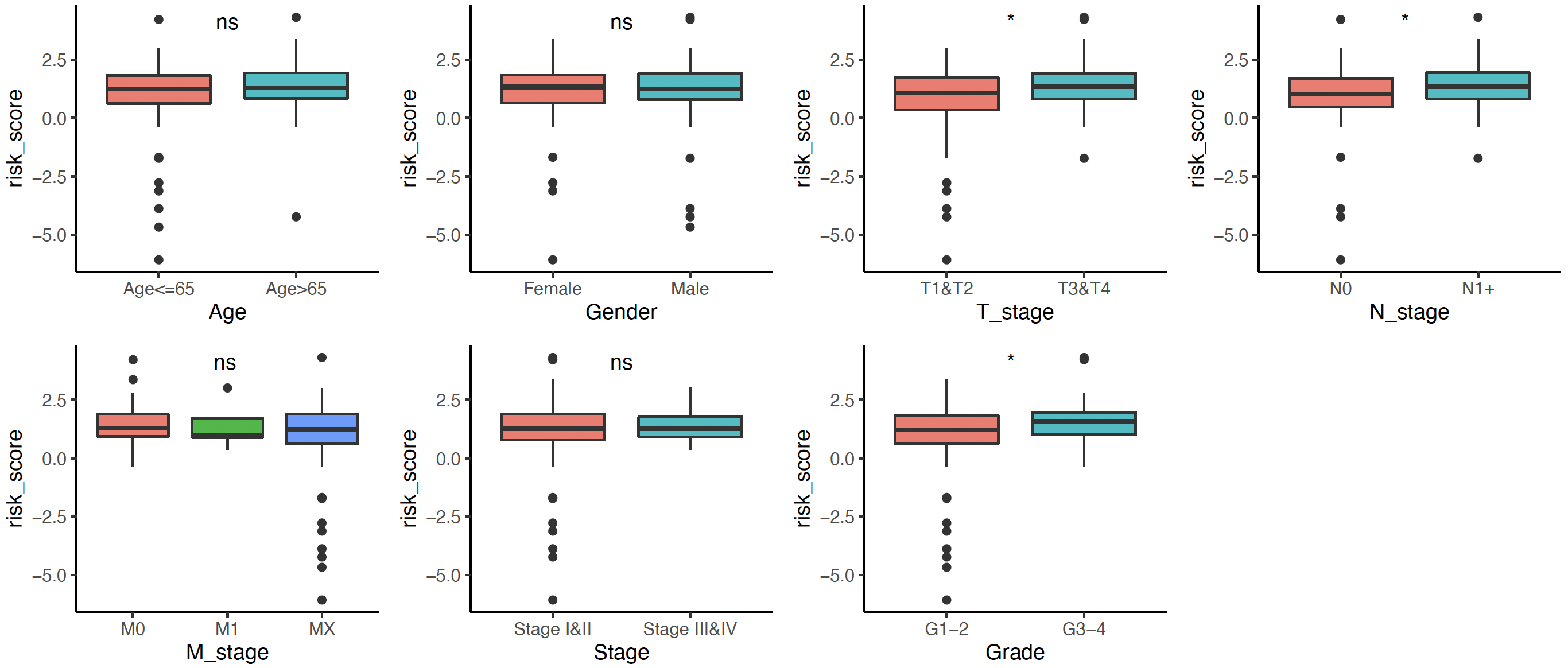
**

**Supplementary Figure 1.** Difference in the AMRS score between different clinicopathologic characteristics.


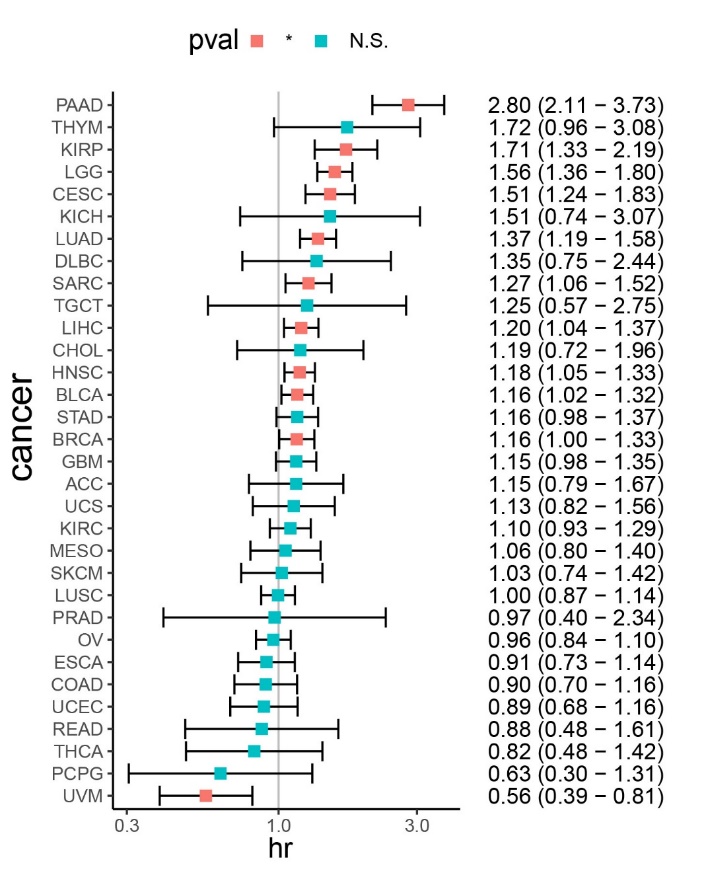


**Supplementary Figure 2.** The correlation between OS and AMRS in TCGA pan-cancer cohorts.


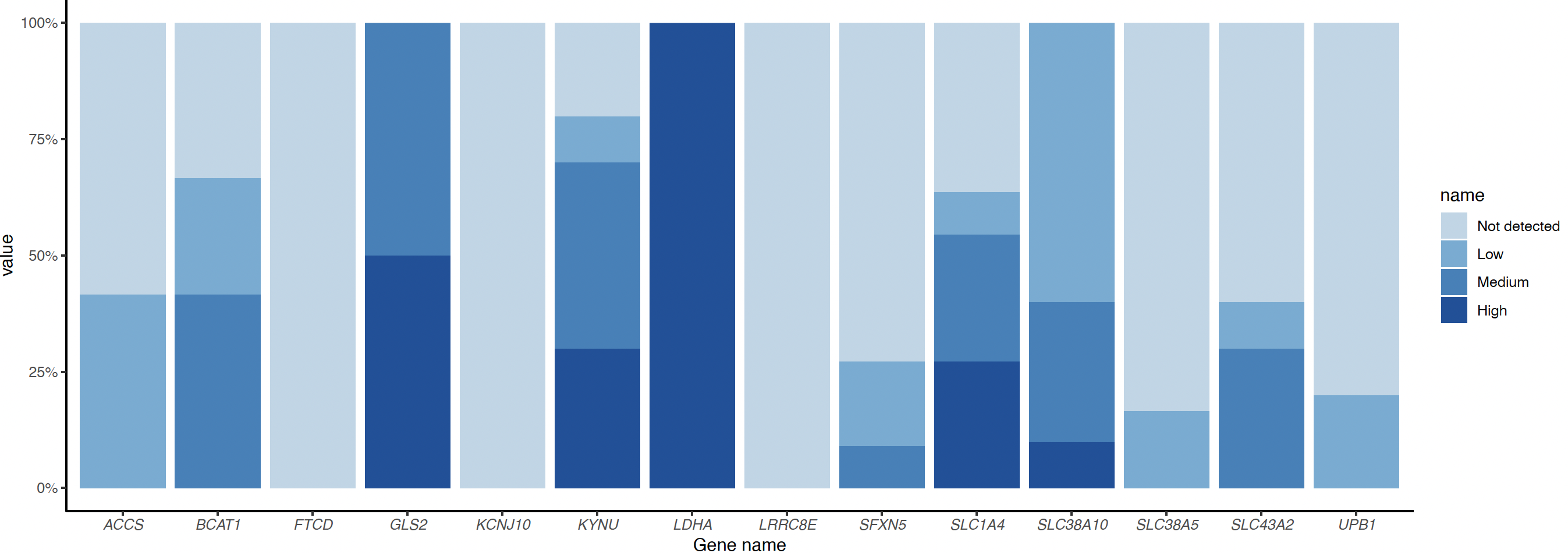


**Supplementary Figure 3.** The protein expression feature of AMRS-related signature genes in pancreatic cancer tissues from human protein atlas.


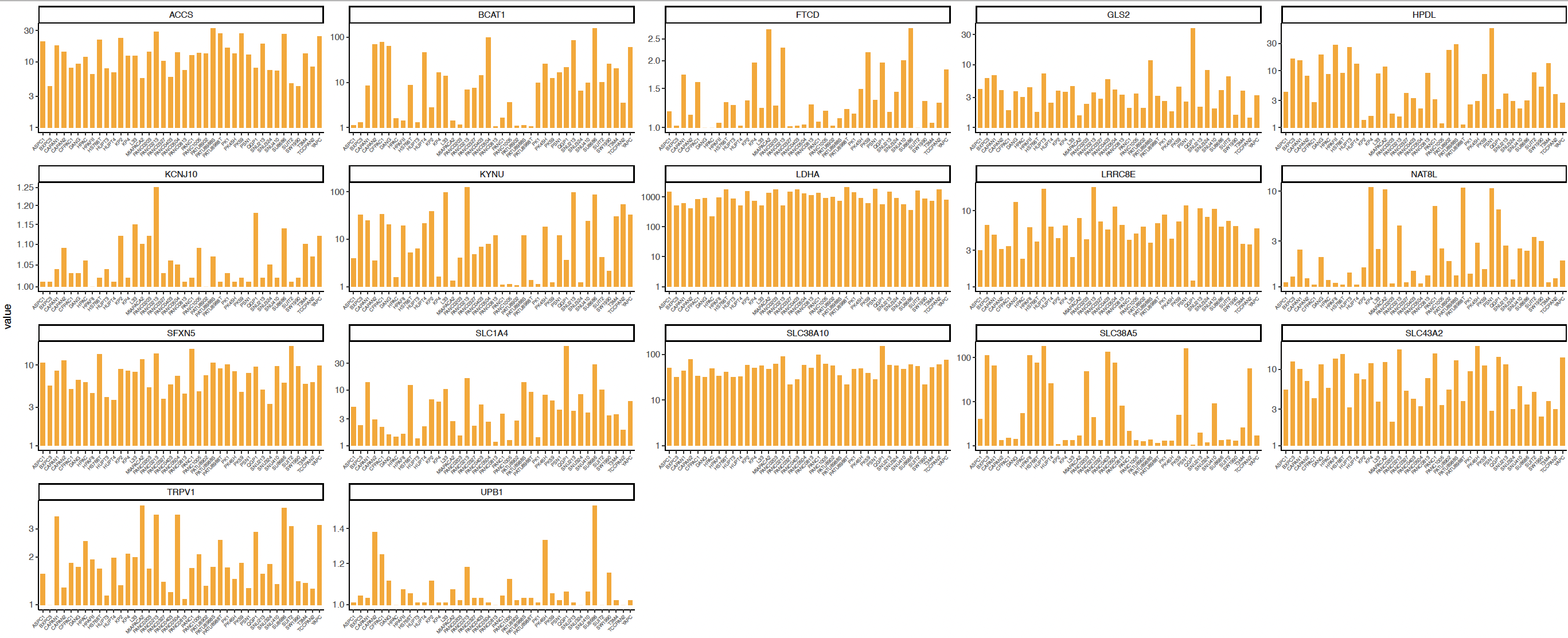


**Supplementary Figure 4.** Difference in the mRNA expression level of AMRS-related signature genes in pancreatic cancer cell lines in CCLE database.


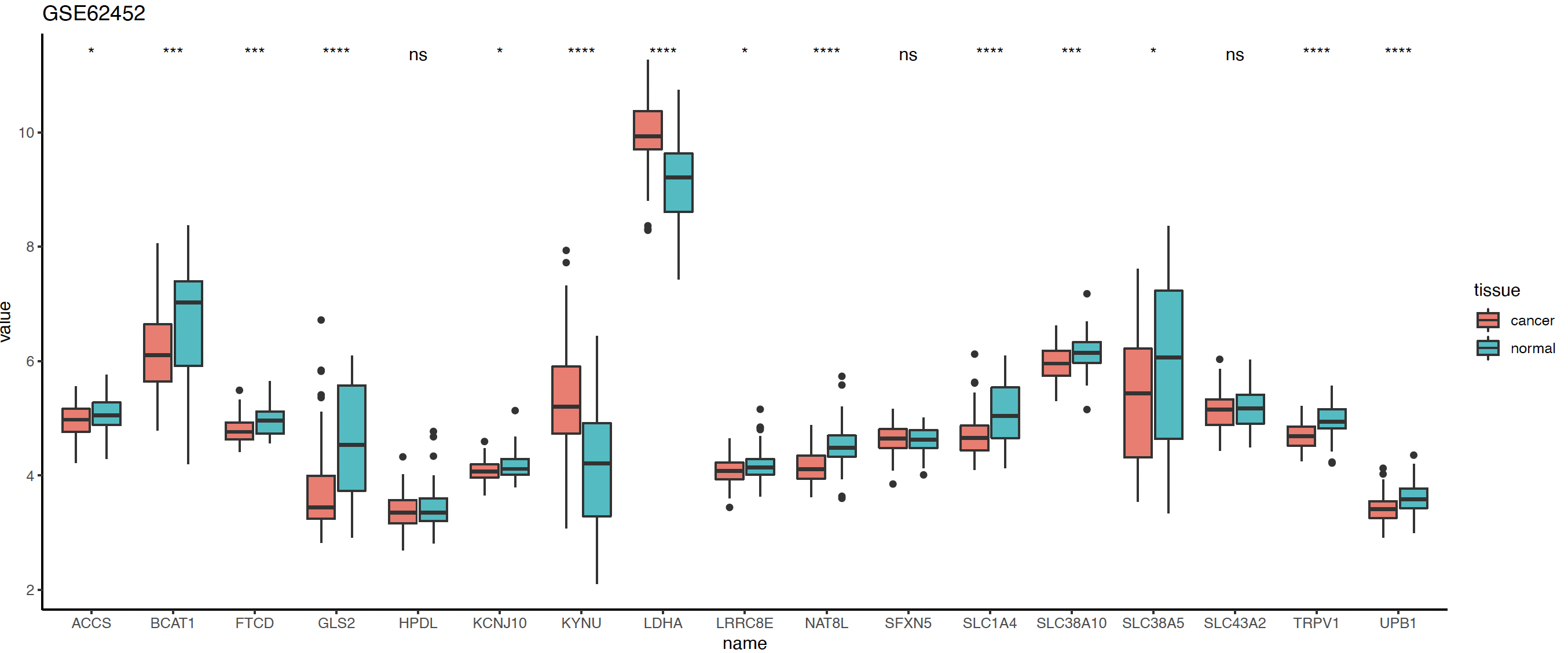


**Supplementary Figure 5.** Difference in the mRNA expression level of AMRS-related signature genes between pancreatic cancer tissues and normal tissues in GSE62452 dataset.
